# Supplementary material for: A direct excitatory projection from entorhinal layer 6b neurons to the hippocampus contributes to spatial coding and memory
Source: Nat Commun. 2022 Aug 16;13:4826. doi: 10.1038/s41467-022-32559-8 (PMC9381769; doi:10.1038/s41467-022-32559-8)
Supplement: Supplementary file 1 — Supplementary Information [file 41467_2022_32559_MOESM1_ESM.pdf]

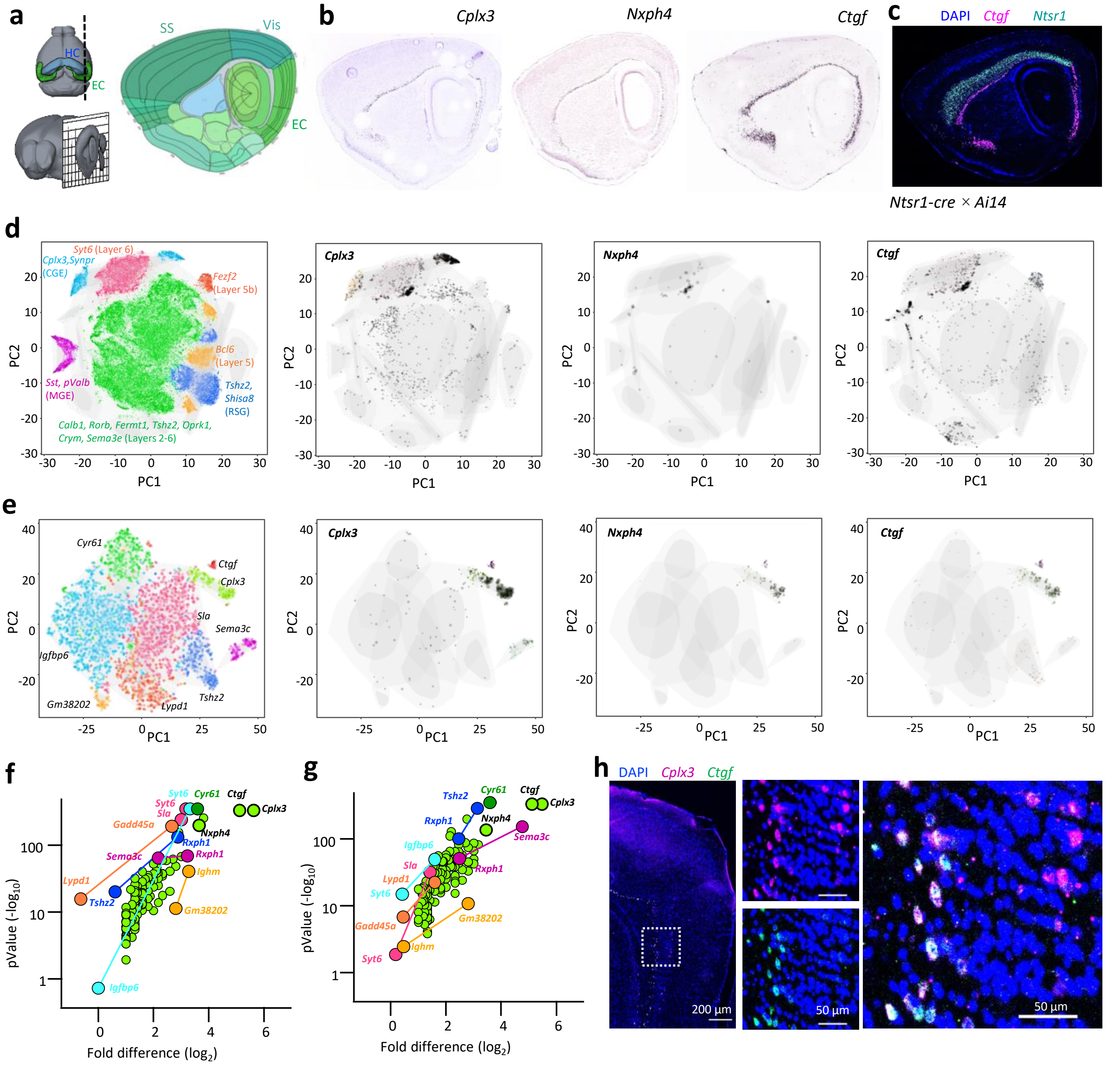


**Supplementary Figure 1 | Molecular identity of *Cplx3*+ neurons in the adult posterior cortex. a,** Three-dimensional schematic representation of the sagittal sectioning plane used in the representative confocal images of this figure. Top schematics were adapted from the Allen Brain Explorer2™ and the bottom image from the Allen common coordinates atlas of the adult mouse brain. Vis – Visual cortex, SS – Somatosensory cortex. **b,** Representative *in situ* hybridization and images of subplate specific markers *Cplx3, Nxph4* and *Ctgf*, showing expression along the innermost cortical layer. **c,** A representative fluorescent *is situ* hybridization (FISH) image of *Ctgf* alongside tdTomato, in the layer 6 marker *Ntsr1*. **d,** tSNE plot of the main neuronal clusters in the posterior cortex (left) and for cells with high *Cplx3, Nxph4* and *Ctgf* expression. Marker genes (italic) and putative identity (parentheses) are noted by each cluster. **e,** Same as (d), but for the sub-clusters comprising the layer 6 cluster, showing that all three genes are enriched within the same sub-cluster. **f,g,** Volcano plots showing the P Value (Barnard's exact test, Bonferroni-adjusted) of genes enriched within the *Cplx3* sub-cluster, in comparison with the entire posterior cortex (f) or with the layer 6 cluster (g), as a function of the difference in mRNA levels, indicating the quality of the gene as a putative selection marker. In both plots, the best marker gene for each of the other sub-clusters in layer 6 is denoted, tethered to the values of the best marker gene of the same sub-cluster, calculated in the neighboring plot. **h,** FISH labeling of *Cplx3* and *Ctgf* mRNA in the EC demonstrates expression colocalization of both genes in EC-6a, but not 5a. Images in panels (a) were adapted from the Allen Mouse Brain Atlas (http://mouse.brain-map.org/) using the following experiments: Cplx3 – 68795395; Nxph4 – 71234703; Ctgf – 79632240. Images in panel (b) was adapted from the Allen Mouse Brain transgenic characterization tool (http://connectivity.brain-map.org/transgenic/) experiment# 113103581. Data source for data in panels (c–f) - Dropviz.org

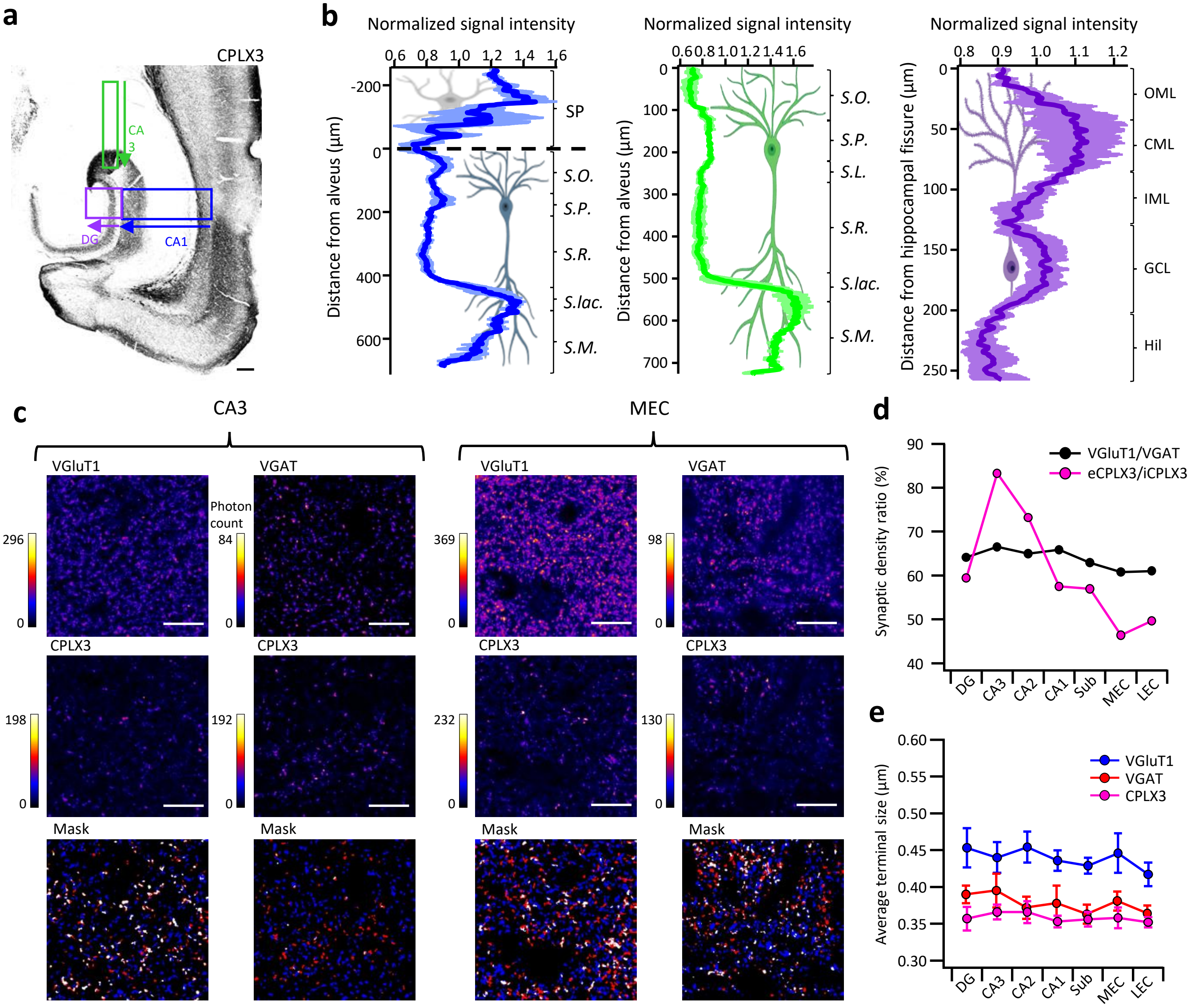


**Supplementary Figure 2 | Localization and differential expression of CPLX3 along the cortico-hippocampal molecular layer. a,** Representative horizontal section stained for CPLX3. Rectangles indicate the extent of the sampled region as well as the sampling direction. Scale bar indicates 200 µm. **b,** Signal intensity was measured and normalized for each of the regions indicated in (a) along the basal-to-apical axis (N = 3 sections from 3 different animals). Each plot represents the averaged normalized fluorescence values for the CA1 (left), CA3 (middle), and DG (right), showing peak fluorescence at the *S.LM.* of both CA3 and CA1 and the CML of the DG. **c,** Representative STED images of VGluT1/VGAT (top row) and CPLX3 (middle row) from the CA3 or MEC *S.M.* Analysis of colocalization between the synaptic markers demonstrates interregional differences in eCPLX3 and iCPLX3 density (bottom row, blue – VGluT1/VGAT, Red – CPLX3, White – Overlay). Scale bars represent 10 µm. **d,** Summary plot of the ratio between eCPLX3 and iCPLX3 synaptic density in each region, in comparison with the ratio between VGluT1 and VGAT density. **e,** Average terminal size for each marker in each region, calculated as the signal area divided by the number of individual spots, show no interregional differences for each marker. All data shown as mean and SEM. Abbreviations: SP – subplate; *S.O. – Stratum oriens; S.P. – Stratum pyramidale; S.R. – Stratum radiatum; S.Lac. – Stratum lacunosum; S.M. – Stratum moleculare; S.L. – Stratum lucidum;* OML – outer molecular layer: CML – central molecular layer; IML – inner molecular layer: GCL – granule cell layer; Hil – hilus.

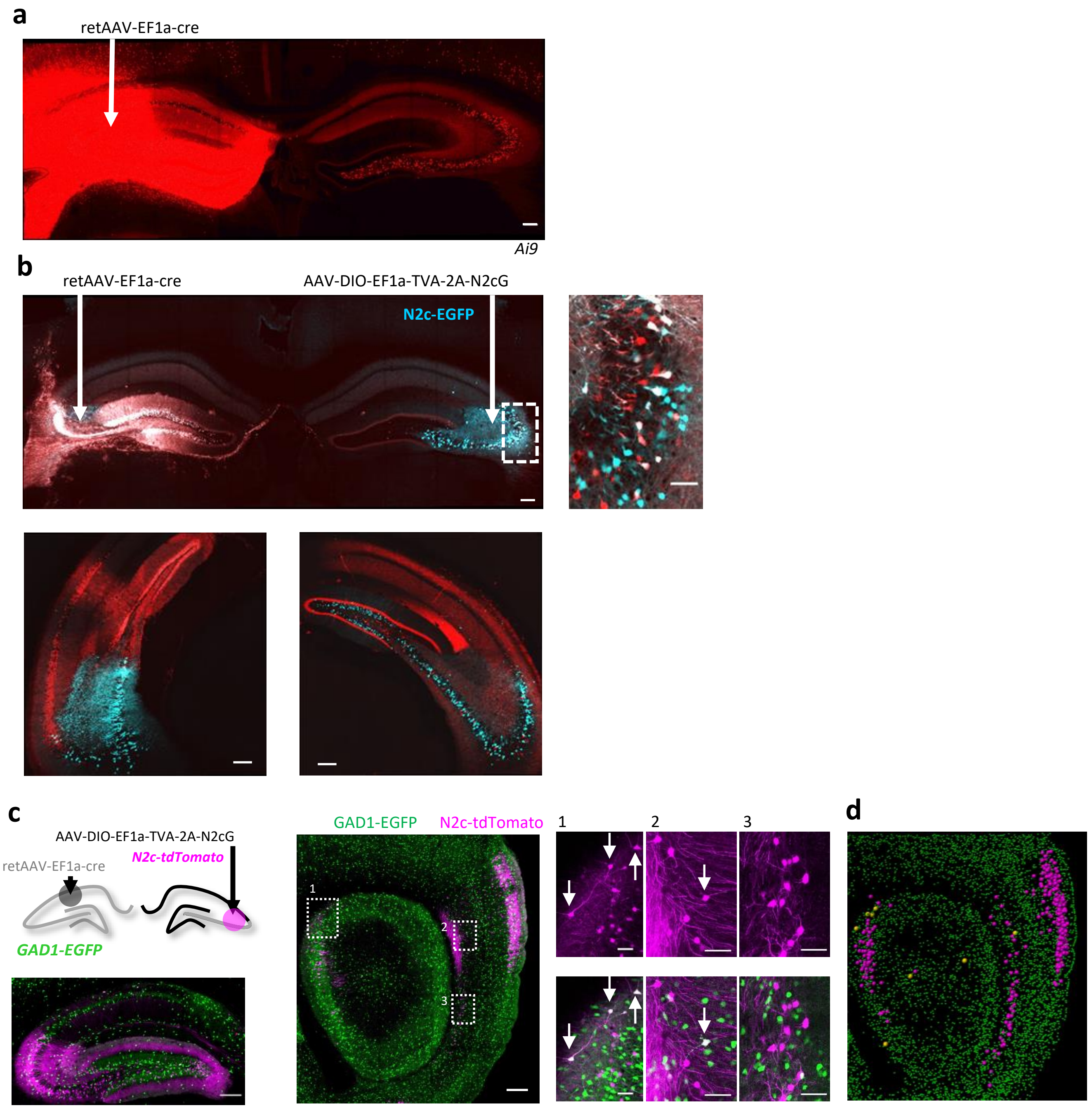


**Supplementary Figure 3 | Specific targeting of hippocampal CA3 for trans-synaptic retrograde labeling. a,** Intracranial injection of concentrated retAAV-EF1a-cre particles to the CA3 of the cre-reporter line Ai9 results in efficient and specific retrograde labeling of CA3, as well as CA2 and hilar mossy cells in the ipsilateral hemisphere. **b,** Targeting the CA3 for retrograde labeling using injection of a cre-dependent AAV expressing the TVA receptor and the N2c glycoprotein, separated by a 2A peptide, along with the retrograde cre injection to the contralateral hemisphere and followed by injection of G-deleted, envA-pseudotyped N2c-EGFP rabies viral vectors. Robust and specific labeling of the CA3 in both hemispheres can be observed along with sparse labeling of dentate granule cells only in the ipsilateral hemisphere to the rabies injection (bottom panels). **c,** Representative sagittal sections of the HC (left) and EC (center) showing cells both in the cortex and the medial hippocampus (right, expanded panels), following retrograde labeling (top left scheme). White arrows in the expanded images to the right are putative CA3-projecting inhibitory neurons. **d,** Analysis panel of the image in (c) used for quantification of inhibitory projections to the CA3. Yellow circles represent cells expressing both tdTomato and EGFP. Scale bars represent 200 µm for full-scale, and 50 µm for expanded insets.

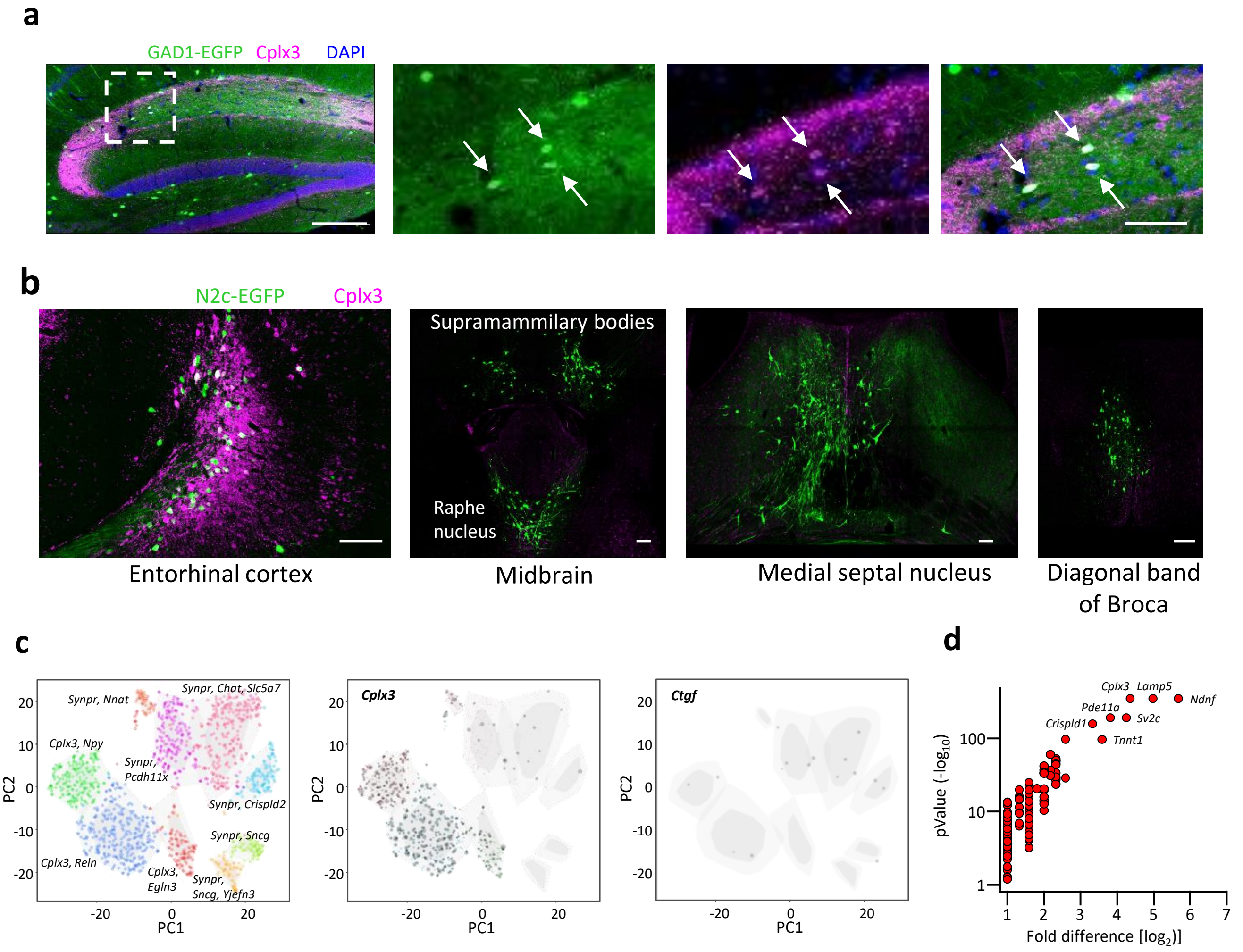


**Supplementary Figure 4 | CPLX3-positive interneurons as a possible source for iCPLX3 synapses in the hippocampal *S.M*. a,** Staining for CPLX3 in horizontal hippocampal sections taken from a Gad1-EGFP mouse. CPLX3$^+$ somata colocalized with EGFP are readily found in the CA1 *S.M.* (white arrows), indicating a potential source of iCPLX3 terminals in this layer. Scale bars indicate 200 µm for full scale, and 50 µm for expanded images. **b,** Confocal images of sub-cortical projections to the CA3, immunolabeled for the subplate-specific marker CPLX3. No overlap could be found for any other region outside of the cortical subplate, confirming it is the only possible source for eCPLX3 positive synaptic terminals in the hippocampal *S.M*. Scale bars indicate 50 µm. **c,** tSNE plot of the main interneuronal clusters in the posterior cortex, with marker genes (italic) noted by each cluster. **d,** tSNE plots for the clusters shown in (c), demonstrating distribution pattern for *Cplx3* (left) and *Ctgf* (right) expressing cells. **e,** Volcano plot showing the genes enriched within the *Cplx3*$^+$ sub-clusters, in comparison with the entire population of interneurons in the posterior cortex. Data source - Dropviz.org.

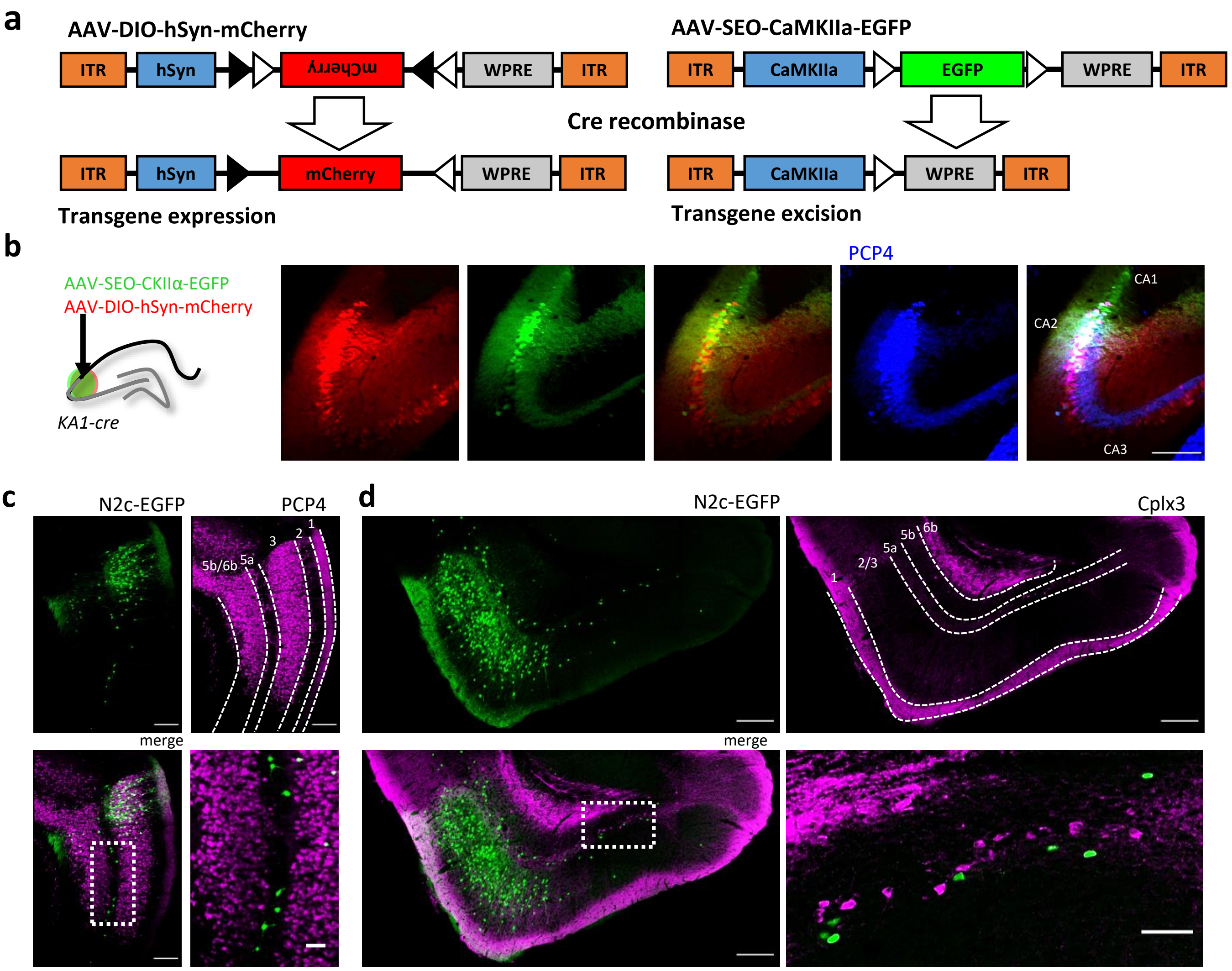


**Supplementary Figure 5 | Specific targeting of CA1 for retrograde labeling. a,** Schematic diagram illustrates the differences in vector structure for the cre-on AAV-DIO and the cre-off AAV-SEO cassettes. **b,** Dual injection of a cre-on vector expressing mCherry under a constitutive promoter, and a cre-off vector expressing EGFP under the excitatory CaMKIIa promoter, into the CA2 sub-region of a KA1cre mouse. EGFP and mCherry expression is largely non-overlapping and divides the CA2 sub-region (indicated by the CA2-specific marker PCP4, blue) into two, relatively equal halves. Scale bar indicates 200 µm. **c,** Representative parasagittal sections, following retrograde labeling from the CA1 using N2c-EGFP, and staining for PCP4 which selectively labels EC-3 and EC-5b, shows a population of neurons along the unlabeled strip between the two labeled cell layers, corresponding to EC-5a. **d,** Representative images of a horizontal section, following retrograde labeling from the CA1 using N2c-EGFP and staining for CPLX3 indicate that CA1-projecting EC-5a neurons do not overlap with the small population of CPLX3-positive neurons which, can be found in layer 5a of the MEC and subicular complex. This lack of overlap suggests that the only CPLX3-positive neurons projecting to the CA1 derive from layer 6b and not from layer 5a. This experiment has been replicated in 5 sections from 3 individual animals. Scale bars represent 200 µm for full scale, and 50 µm for expanded images.

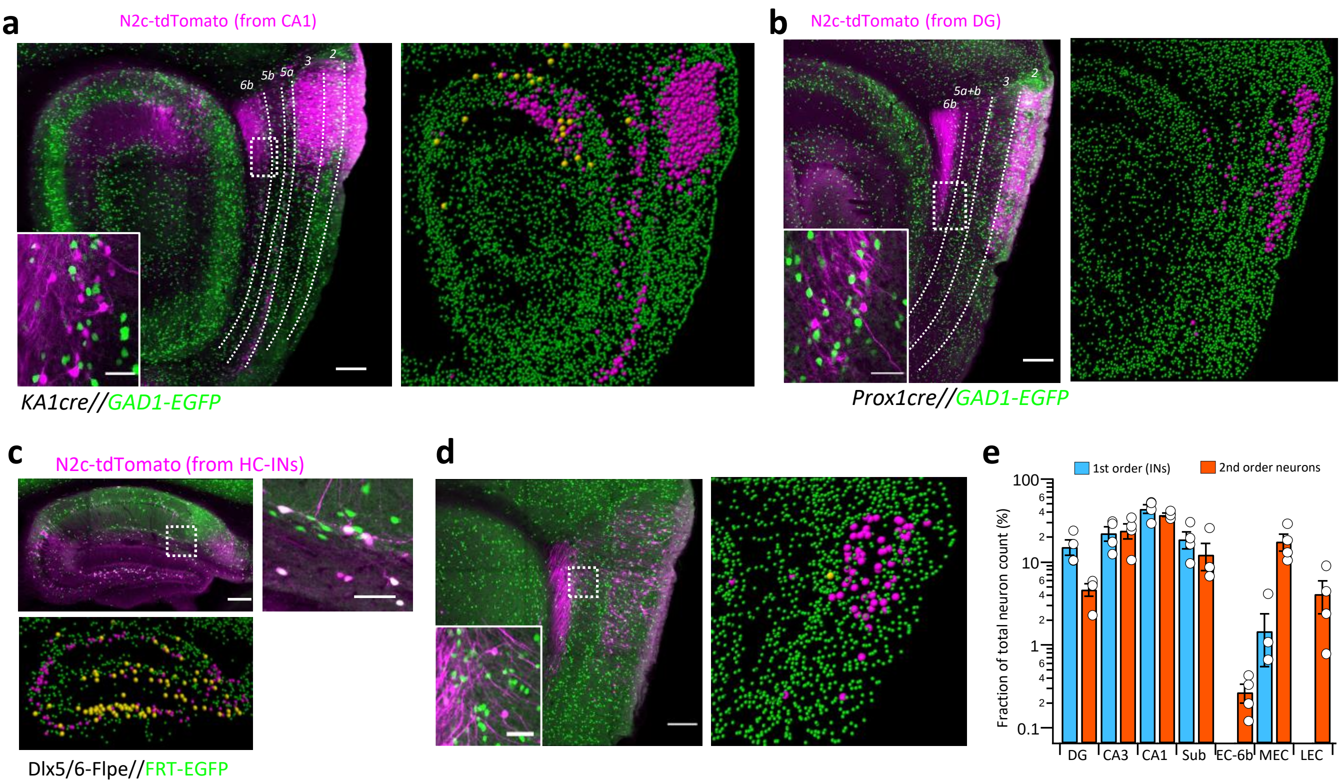


**Supplementary Figure 6 | Long-range inhibitory projections to hippocampal populations. a,b** Retrograde labeling from the CA1 (a) or DG (b) following the approaches described in Fig. 2d and f, respectively, along with specific IN labeling using GAD1-EGFP mice. Both show a near-complete absence of inhibitory projection neurons in the EC-6b, comparable with the projection to the CA3 (Supplementary Fig. 3c). The images selected show the only long-range inhibitory neurons found to project to these regions. **c,** A representative confocal image of the dorsal hippocampus (top) and corresponding analysis panel (bottom) following retrograde labeling from hippocampal INs as in Fig. 2i, demonstrating that a large proportion of the putative starter cells (yellow) are located within the molecular layer. **d,** A representative confocal image of the EC (left) and corresponding analysis panel (right) reveal very sparse labeling from the EC-6b. **e,** Averaged proportions of 1$^{st}$ order INs (tdTomato + EGFP) and their 2$^{nd}$ order projections (tdTomato only) in the tested regions. Bars represent mean and SEM and empty circles represent individual experiments. Scale bars indicate 200 µm for full scale, and 50 µm for expanded images.

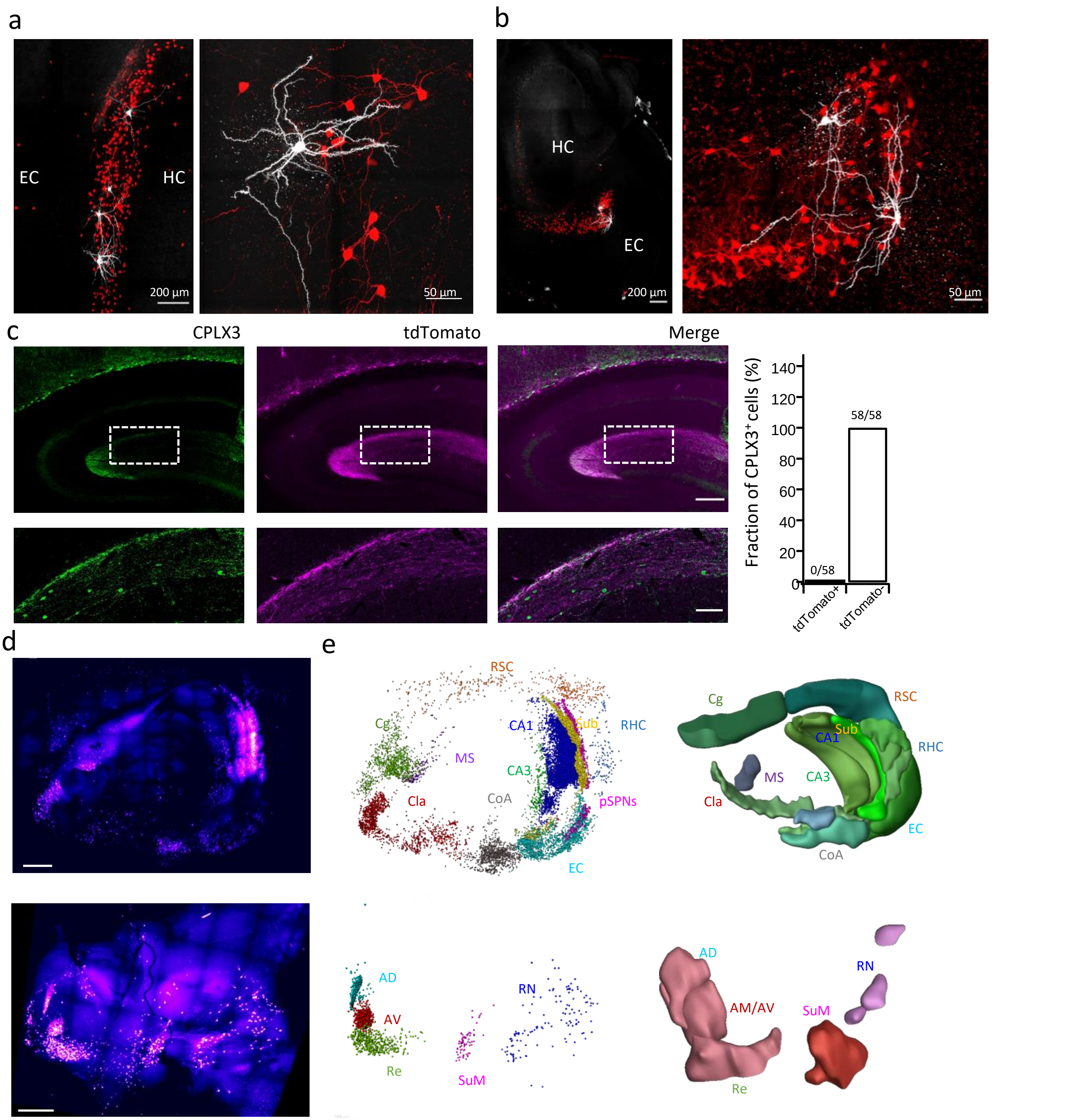


**Supplementary Figure 7 | Specificity of the Ctgf-2A-dgCre line and use for quantification of brain-wide projections to EC-6b neurons. a,b** Representative confocal collapsed image stacks showing sequentially patched, biocytin-filled EC-6b neurons (white) in an acute sagittal (a) or horizontal (b) section of Ctgf-2A-dgCre//Ai9 (red) mice. **c,** Representative confocal images of the hippocampus of Ctgf-2A-dgCre × Ai9 mouse, following immunolabeling for CPLX3 confirms colocalization of the two markers in the cortical layer 6b (top) but not in Cplx3$^{+}$ interneurons of the CA1 molecular layer (bottom) along with quantification of the fraction of CPLX3$^{+}$ *S.LM.* Interneurons, also expressing tdTomato (right, N = 58 cells/9 sections/3 animals). Scale bars represent 200 and 50 μm for the top and bottom images, respectively. **d,** Representative 2D images of a 3D image stack of the intact, clarified cortical plate (top) or brainstem (bottom) tissue following retrograde labeling from the EC-6b using N2c-EGFP. Scale bars represent 800 μm **e,** Analysis panels corresponding to the images in (c), demonstrating classification of 2$^{nd}$ order projection neurons in the cortical plate (top left) and brainstem (bottom left). 1$^{st}$ order neurons were identified by co-expression of tdTomato, either from Ctgf-2A-dgCre × Ai9 or AAV-DIO-Ef1a-tdTomato, injected alongside the TVA-2A-N2cG cassette. Images to the right of the analysis panels were adapted from the Allen Institute's the Brain Explorer 2™ and provide reference to the location of the different regions in the mouse brain.

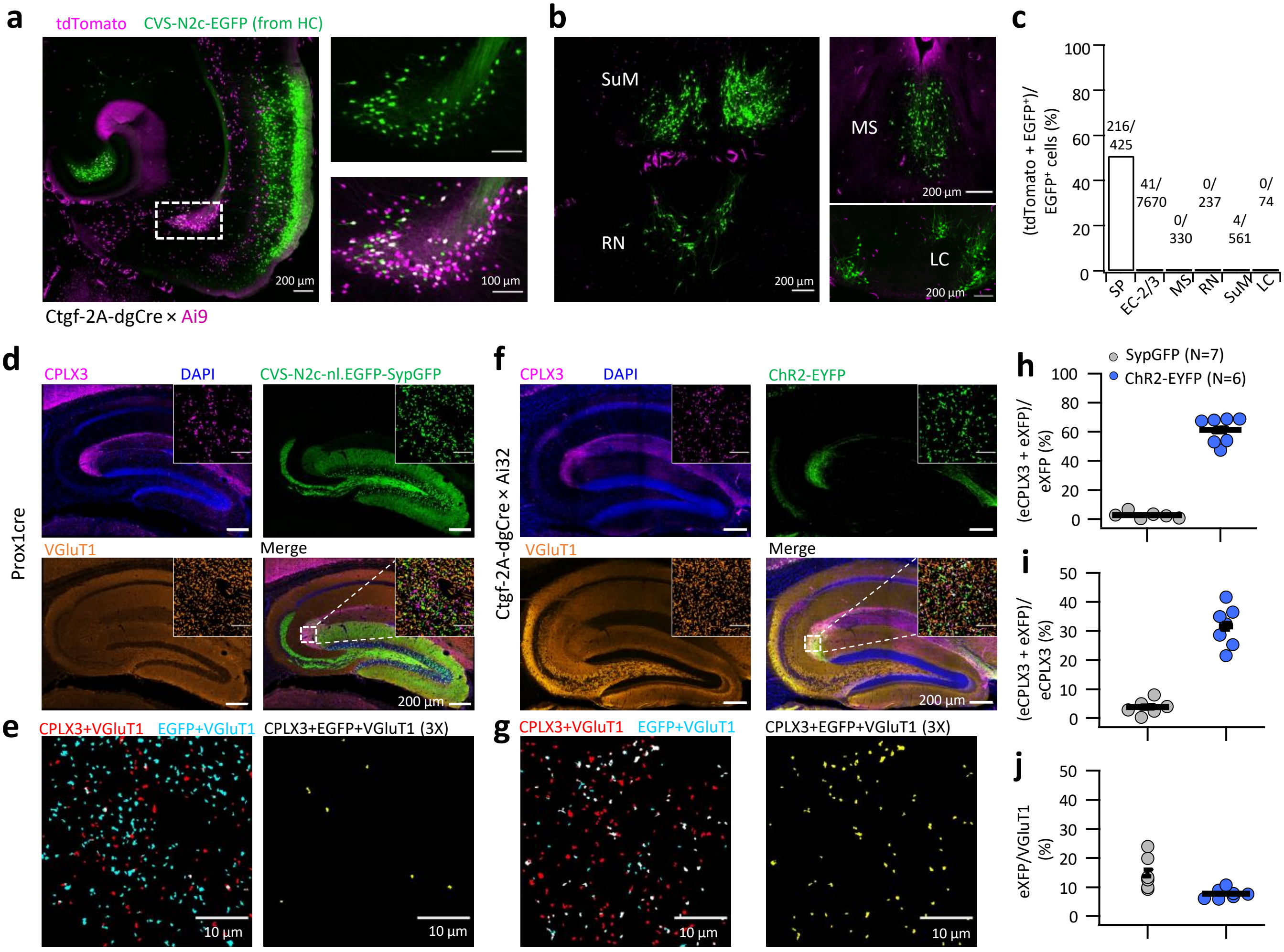


**Supplementary Figure 8 | Labeling specificity of the EC-6b to CA3 pathway for optogenetic stimulation. a,b,** Representative confocal images of the horizontal plane following non-specific retrograde labeling from the hippocampus of Ctgf-2A-dgCre crossed with Ai14 mice, using native-coat RVdG-CVS-N2c-EGFP, to label hippocampal afferents in the EC (a) as well as subcortical regions (b), along with *Ctgf*$^+$ cells. **c,** Relative proportions of double labeled EGFP$^+$ tdTomato$^+$ cells, as a fraction of the total EGFP$^+$ count. Number of cells with overlapping signal of the total number of EGFP$^+$ cells per region counted from 3 separate animals is shown above the respective bar. **d,** Representative confocal images of the sagittal plane of a prox1-cre mouse, following DG-specific retrograde labeling using RVdG$_{envA}$-CVS-N2c-nl.EGFP-SypEGFP vectors, immunolabeled for CPLX3 and VGluT1. Insets on the top right-hand corner of each image show a mask of the signal for each channel, acquired using combined STED (CPLX3 and VGluT1) and confocal (EGFP) microscopy. **e,** Masks of the overlay between VGluT1 and either CPLX3 (red, left) or EGFP (cyan, left) and between all three channels (CPLX3+EGFP+VGluT1=3X). **f,g,** Same as (d,e) but for a Ctgf-2A-dgCre crossed with Ai32 mise. **h,i,** Quantification of the relative synaptic density of triple-labeled synapses as a fraction of excitatory CPLX3$^+$ (eCPLX3) (h) or excitatory EGFP$^+$ or EYFP$^+$ (eXFP) synapses (i). **j,** Relative fraction of the eXFP$^+$ synaptic density, as a fraction of the entire VGluT1 signal, indicates the relative fraction of SypGFP/ChR2-EYFP of the total pool of excitatory synapses in the *S.M.* of the CA3. Number of individual sections imaged from 3 separate animals is indicated in the legend. Data in h-j shown as individual data points, with mean and SEM shown as horizontal and vertical black lines.

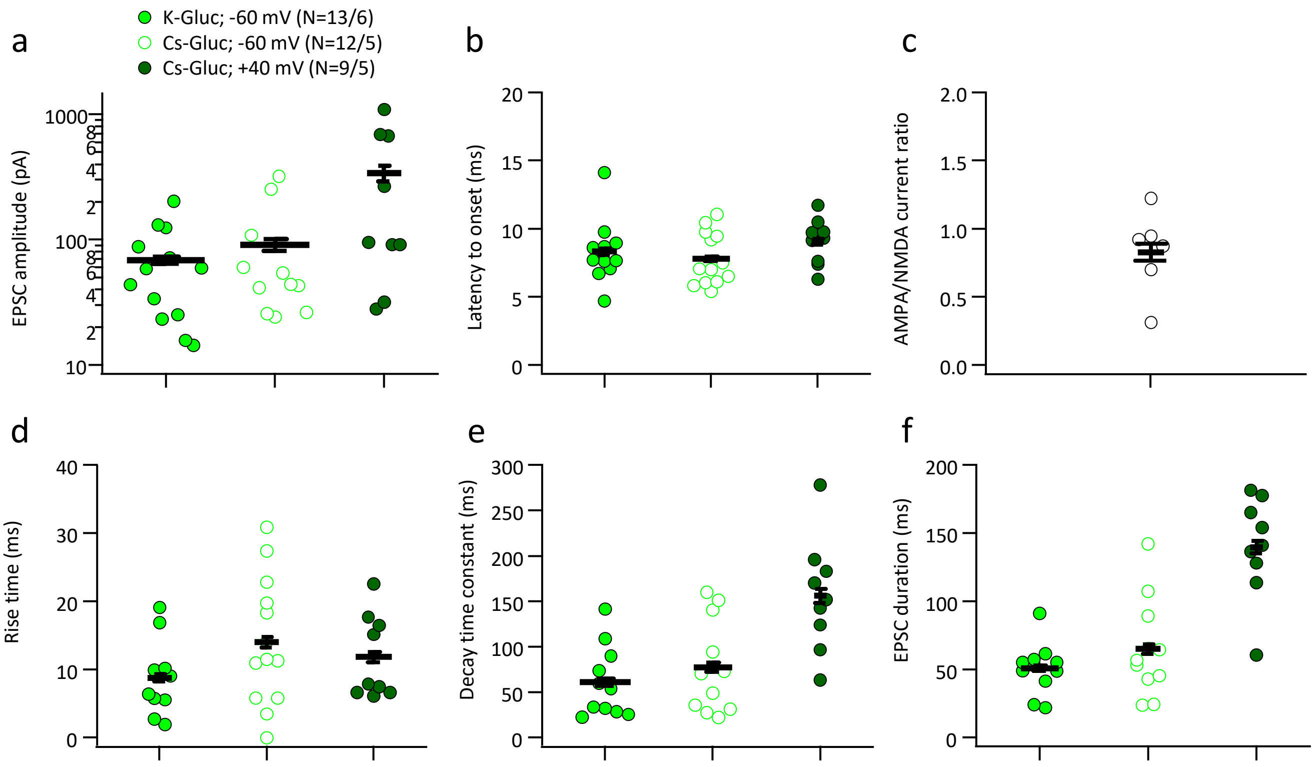


**Supplementary Figure 9 | Optogenetically-evoked SP-CA3 responses are primarily mediated by AMPA and NMDA receptors. a–f,** Summary plots comparing the EPSC properties for the EC-6b CA3 projection, under different recording conditions designed to separate AMPA receptor-mediated currents (−60 mV) and NMDA receptor-mediated currents (+40 mV): peak amplitude of the first response (a), latency to EPSC onset from the start of the stimulation (b), 20–80% rise time (c), decay time constant (d), duration at half maximum (e) and AMPA/NMDA peak current ratio (f). Individual data points are shown as circles, horizontal and vertical black lines denote mean and S.E.M. Number of cells/animals from each condition shown in parentheses in the legend.

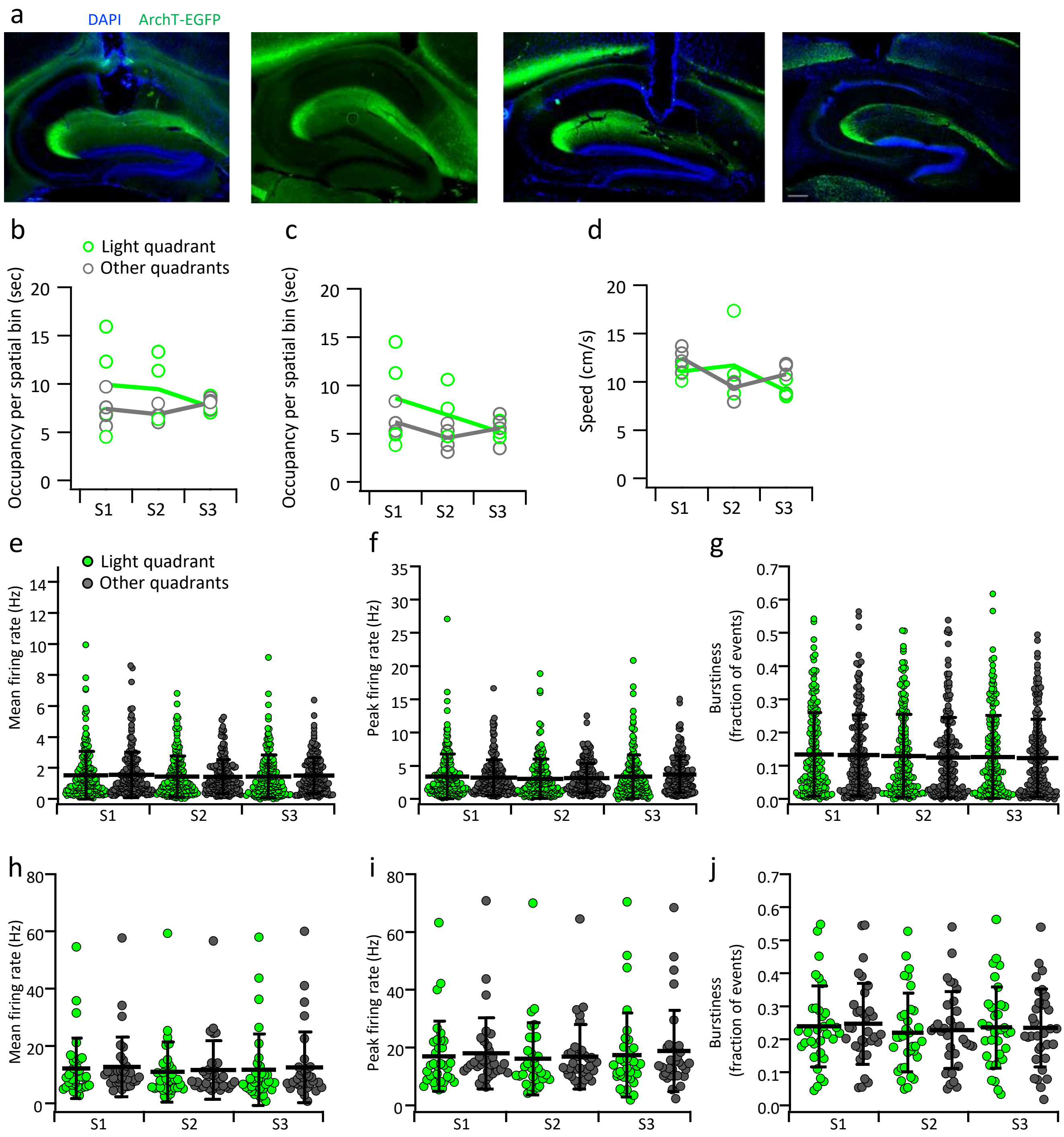


**Supplementary Figure 10 | Animal locomotion and place-cell spiking parameters across recording sessions. a,** Representative images of fiber implantation location for each of the four experimental animals. **b–d,** Comparison of average total occupancy time (b) or only time above the speed criterion (c) and average locomotion speed (d) between the target quadrant (green) and the rest of the arena (gray), across the three sessions. Data for the individual animals is denoted by open circles and the average is denoted by a continuous line **e–g,** A comparison of pyramidal cells' mean firing rate (e), peak firing rate (f) and the fraction of bursts (spikes with <10 ms interspike interval) of total spike count (f), between the target quadrant and the rest of the arena of putative pyramidal neurons across the three sessions (n = 183 cells from 4 animals). **h–j,** Same comparisons as (e–g), but for inhibitory neurons (n = 33 cells from 4 animals). Horizontal and vertical black lines denote mean and SD. No statistically significant differences found across conditions and sessions in any of the comparisons using a two-sided Kruskal-Wallis test (Holm-Bonferroni adjusted).

Supplementary Table 1: Summary of pSPN membrane properties.

| **Property**[1,2] | **Mean** | **SEM** |
|---|---|---|
| Resting membrane potential (mV) | -68.7345 | 1.18683 |
| AP threshold potential (mV) | -48.1541 | 1.39943 |
| AP peak[3] (mV) | 83.34582 | 2.15426 |
| AP rise time[4] (ms) | 0.605888 | 0.04134 |
| AP width[5] (ms) | 1.513645 | 0.04891 |
| Max rise slope | 230.1616 | 9.25946 |
| Max decay slope | 137.6243 | 4.31897 |
| Afterhyperpolarization[6] (mV) | 11.54226 | 1.40138 |
| Latency to first AP (ms) | 303.2677 | 38.3723 |
| Input resistance (MΩ) | 487.4063 | 23.1846 |
| Sag ratio[7] (%) | 5.8414 | 1.281 |

1. N=31 cells.
2. For each cells, all AP properties were measured for the first AP at the rheobase current.
3. Measured from 0.
4. 20%-80% of peak.
5. Full-width at half-maximum.
6. Relative to the threshold potential.
7. Measured as the ratio between the membrane potential at the positive peak at the start of the response to a -100 pA current injection, 1 s long, in relation to the average potential at the final 100 ms before the end of the pulse.

Supplementary Table 2: Summary of plasmids.

| Plasmid name | Source (deposited by) | Cat# |
|---|---|---|
| pCAG-B19N | AddGene (I. Wickersham) | #59924 |
| pCAG-B19P | AddGene (I. Wickersham) | #59925 |
| pCAG-B19L | AddGene (I. Wickersham) | #59922 |
| pAdDeltaF6 | AddGene (J. Wilson) | #112867 |
| rAAV-DJ RepCap | Gift from Mark A. Kay | |
| rAAV2-retro helper | AddGene (A. Karpova and D. Schaffer) | #81070 |
| pAAV-DIO-Ef1a-TVA-2A-N2cG | AddGene (Y. Ben Simon and P. Jonas) | #172360 |
| pAAV-EF1a-Cre | AddGene (K. Deisseroth) | #55636 |
| pAAV-SEO-CaMKIIa-TVA-2A-N2cG | AddGene (Y. Ben Simon and P. Jonas) | #172363 |
| pAAV-FRT-Ef1a-TVA-2A-N2cG | AddGene (Y. Ben Simon and P. Jonas) | #172361 |
| pAAV-SEO-CaMKII-EGFP | AddGene (Y. Ben Simon and P. Jonas) | #172361 |
| pAAV-DIO-hSyn-mCherry | AddGene (K. Deisseroth) | #114472 |
| RVdG-CVS-N2c-EGFP | AddGene (T. Jessell) | #73461 |
| RVdG-CVS-N2c-tdTomato | AddGene (T. Jessell) | #73462 |
| RVdG-CVS-N2c-tdTomato-ChIEF | AddGene (Y. Ben Simon and P. Jonas) | #172370 |
| RVdG-CVS-N2c-nl.EGFP-SypGEFP | AddGene (Y. Ben Simon and P. Jonas) | #172380 |

Supplementary Table 3: Summary of transgenic lines.

| **Transgenic line** | **Full name** | **Source (deposited by)** | **Cat#** |
|---|---|---|---|
| Ai9 | B6.Cg-Gt(ROSA)26Sor$^{\text{tm9(CAG-tdTomato)Hze}}$/J | Jackson labs (H. Zeng) | 007909 |
| GAD1-EGFP | | K. Obata and Y.Yanagawa | |
| RCE-FRT | Gt(ROSA)26Sor$^{\text{tm1.2(CAG-EGFP)Fsh}}$/Mmjax | Jackson labs (G. Fishell) | 32038 |
| Prox1-cre | Tg(Prox1-cre)SJ32Gsat/Mmucd | MMRRC (N. Heintz) | 036644-UCD |
| KA1-cre | C57BL/6-Tg(Grik4-cre)G32-4Stl/J | Jackson labs (S. Tonegawa) | 006474 |
| Dlx5/6-FlpE | Tg(mI56i-flpe)39Fsh/J | Jackson labs (G. Fishell) | 010815 |
| Ctgf-2A-dgCre | B6.Cg-Ccn2$^{\text{tm1.1(folA/cre)Hze/}}$J | Jackson labs (H. Zeng) | 028535 |
| Ai32 | B6.Cg-Gt(ROSA)26Sor$^{\text{tm32(CAG-COP4*H134R/EYFP)Hze}}$/J | Jackson labs (H. Zeng) | 024109 |
| Ai40 | B6.Cg-Gt(ROSA)26Sor$^{\text{tm40.1(CAG-aop3/EGFP)Hze}}$/J | Jackson labs (H. Zeng) | 021188 |
| RosaDTA | B6.129P2-*Gt(ROSA)26Sor*$^{tm1(DTA)Lky}$/J | Jackson labs (R. Locksley) | 009669 |
