# Supplementary material for: A direct excitatory projection from entorhinal layer 6b neurons to the hippocampus contributes to spatial coding and memory
Source: Nat Commun. 2022 Aug 16;13:4826. doi: 10.1038/s41467-022-32559-8 (PMC9381769; doi:10.1038/s41467-022-32559-8)
Supplement: Supplementary file 3 — Reporting Summary [file 41467_2022_32559_MOESM3_ESM.pdf]

nature research

Corresponding author(s): Yoav Ben Simon

Last updated by author(s): Jul 28, 2022

Reporting Summary

Nature Research wishes to improve the reproducibility of the work that we publish. This form provides structure for consistency and transparency in reporting. For further information on Nature Research policies, see our Editorial Policies and the Editorial Policy Checklist.

Statistics

For all statistical analyses, confirm that the following items are present in the figure legend, table legend, main text, or Methods section.

n/a	Confirmed	
☐	☒	The exact sample size (*n*) for each experimental group/condition, given as a discrete number and unit of measurement
☐	☒	A statement on whether measurements were taken from distinct samples or whether the same sample was measured repeatedly
☐	☒	The statistical test(s) used AND whether they are one- or two-sided<br>*Only common tests should be described solely by name; describe more complex techniques in the Methods section.*
☐	☒	A description of all covariates tested
☐	☒	A description of any assumptions or corrections, such as tests of normality and adjustment for multiple comparisons
☐	☒	A full description of the statistical parameters including central tendency (e.g. means) or other basic estimates (e.g. regression coefficient) AND variation (e.g. standard deviation) or associated estimates of uncertainty (e.g. confidence intervals)
☐	☒	For null hypothesis testing, the test statistic (e.g. *F*, *t*, *r*) with confidence intervals, effect sizes, degrees of freedom and *P* value noted<br>*Give P values as exact values whenever suitable.*
☒	☐	For Bayesian analysis, information on the choice of priors and Markov chain Monte Carlo settings
☒	☐	For hierarchical and complex designs, identification of the appropriate level for tests and full reporting of outcomes
☐	☒	Estimates of effect sizes (e.g. Cohen's *d*, Pearson's *r*), indicating how they were calculated

Our web collection on statistics for biologists contains articles on many of the points above.

Software and code

Policy information about availability of computer code

Data collection	The following data collection scripts, used for the specified purposes, are publicly available:<br>https://git.ist.ac.at/alois.schloegl/ktan.git - acquisition and digitization of raw electrophysiological data from CA1-implanted tetrodes<br>https://github.com/kevin-allen/positrack - digitization of animal location.<br>Igor Pro 6.37 and FPulse version 3.3.3 (U. Fröbe, Physiological Institute, University of Freiburg, Germany) - pulse generation and in vitro electrophysiological data acquisition.<br>Intellicage controller (TSE-system, Germany) - acquisition of intellicage behavioral data
Data analysis	The following data analysis scripts, used for the specified purposes, are publicly available:<br>https://github.com/sommerc/coloco3surf - Analysis of signal overlay in STED images.<br>https://github.com/igridchyn/lfp_online - Clustering of sorted spikes into single-units.<br>ImageJ 1.53q - image analysis.<br>Stimfit 0.15.8 - analysis of in vitro electrophysiological data.<br>Imaris 9.2 (Oxford instruments) - image analysis.<br>Python 3 - place cell analysis<br>R 4.1.0 - statistical analysis.<br>Octave 6.2.0 - statistical analysis.<br>Intellicage analyzer (TSE-system, Germany) - analysis of intellicage behavioral data.

For manuscripts utilizing custom algorithms or software that are central to the research but not yet described in published literature, software must be made available to editors and reviewers. We strongly encourage code deposition in a community repository (e.g. GitHub). See the Nature Research guidelines for submitting code & software for further information.

Data

Policy information about availability of data

All manuscripts must include a data availability statement. This statement should provide the following information, where applicable:

- Accession codes, unique identifiers, or web links for publicly available datasets
- A list of figures that have associated raw data
- A description of any restrictions on data availability

All datasets generated in this study are available in the source data file.
For representative images from the Allen Brain Atlas used in Extended Data Figure 1 the following experiments were used:
Cplx3 - 68795395
Nxph4 - 71234703
Ctgf - 79632240
Ctgf + Ntsr1 (FISH) - 113103581

Field-specific reporting

Please select the one below that is the best fit for your research. If you are not sure, read the appropriate sections before making your selection.

[x] Life sciences [ ] Behavioural & social sciences [ ] Ecological, evolutionary & environmental sciences

For a reference copy of the document with all sections, see nature.com/documents/nr-reporting-summary-flat.pdf

Life sciences study design

All studies must disclose on these points even when the disclosure is negative.

Sample size	No statistical methods were used to predetermine sample sizes. Sample numbers for intellicage experiments were based on a similar work with a similar experimental design (Voikar et al., 2018).
Data exclusions	For behavioral intellicage experiments, Animals which failed to attain failure rates lower than 70 % by the last day of the familiarization phase were excluded from the final analysis. This exclusion criterion was pre-established based on results of a probe experiment with WT mice. One mouse was excluded from the analysis of place cell activity after tissue analysis revealed that the optic fiber was placed below the CA1 molecular layer.
Replication	All experiments described in this manuscript can be reproduced.
Randomization	In behavioral intellicage experiments, before being assigned to the experimental or control group, the performance of all animals in the learning phase immediately prior to the manipulation was ranked and the animals were then alternately assigned to either of the two groups, with the best-performing animal always assigned to the experimental group. water ports were allocated randomly prior to manipulation and then alternated in an identical manner for all animals, regardless of condition.<br>For in-vivo electrophysiology experiments, animals were randomly assigned a target corner and each animal was assigned with a different one.<br>For all other experiments, groups consisted of animals from different transgenic lines and therefore they could not be randomly assigned into groups.
Blinding	Behavioral experiments were completely automated and without experimenter involvement.<br>For in-vivo electrophysiology experiments, blinding was not possible as in each condition the fluorescence was differently distributed and stimulation location had to be determined according to the condition.

Reporting for specific materials, systems and methods

We require information from authors about some types of materials, experimental systems and methods used in many studies. Here, indicate whether each material, system or method listed is relevant to your study. If you are not sure if a list item applies to your research, read the appropriate section before selecting a response.

Materials & experimental systems

n/a	Involved in the study
☐	☒ Antibodies
☐	☒ Eukaryotic cell lines
☒	☐ Palaeontology and archaeology
☐	☒ Animals and other organisms
☒	☐ Human research participants
☒	☐ Clinical data
☒	☐ Dual use research of concern

Methods

n/a	Involved in the study
☒	☐ ChIP-seq
☐	☒ Flow cytometry
☒	☐ MRI-based neuroimaging

Antibodies

Antibodies used

CPLX3 (Synaptic Systems, Cat#122302, 1:500)
VGLUT1 (Synaptic Systems, Cat# 135304, 1:500)
VGAT (Synaptic Systems, Cat# 131003, 1:500)
PCP4 (Novus Biologicals, Cat# NBP1-80929, 1:500)
NeuN (Novus Biologicals, Cat# NBP1-92693, 1:1000)
Goat anti-Rabbit IgG (H+L) Cross-Adsorbed Secondary Antibody, Alexa Fluor™ 488 (ThermoFisher, Cat# A-11008, 1:500)
Goat anti-Rabbit IgG (H+L) Highly Cross-Adsorbed Secondary Antibody, Alexa Fluor™ 647 (ThermoFisher, Cat# A-21245, 1:500)
Anti-guinea pig Star635P (Abberior, Cat# ST635P, 1:200)
Anti-rabbit Star580 (Abberior, ST580, 1:200)

Validation

For CPLX3, we ran an additional test for the antibody using several CPLX3 KO mice, generously given to us by Prof. Nils Brose, with results confirming the absence of reactivity in the brain tissue.

Furthermore, anti CPLX3 antibody was validated for mouse by Viswanathan S, Sheikh A, Looger LL, Kanold PO. Molecularly Defined Subplate Neurons Project Both to Thalamocortical Recipient Layers and Thalamus. Cereb Cortex. 2017 Oct 1;27(10):4759-4768. doi: 10.1093/cercor/bhw271. PMID: 27655928; PMCID: PMC6059176.

Anti VGluT1 was validated for mouse by López-Hernández T, Takenaka KI, Mori Y, Kongpracha P, Nagamori S, Haucke V, Takamori S. Clathrin-independent endocytic retrieval of SV proteins mediated by the clathrin adaptor AP-2 at mammalian central synapses. Elife. 2022 Jan 11;11:e71198. doi: 10.7554/eLife.71198. PMID: 35014951; PMCID: PMC8752090.

Anti VGAT was validated for mouse by Yao W, Tambini MD, Liu X, D'Adamio L. Tuning of Glutamate, But Not GABA, Release by an Intrasynaptic Vesicle APP Domain Whose Function Can Be Modulated by β- or α-Secretase Cleavage. J Neurosci. 2019 Aug 28;39(35):6992-7005. doi: 10.1523/JNEUROSCI.0207-19.2019. Epub 2019 Jun 24. PMID: 31235642; PMCID: PMC6733572.

Anti PCP4 was validated for mouse by Kohara K, Pignatelli M, Rivest AJ, Jung HY, Kitamura T, Suh J, Frank D, Kajikawa K, Mise N, Obata Y, Wickersham IR, Tonegawa S. Cell type-specific genetic and optogenetic tools reveal hippocampal CA2 circuits. Nat Neurosci. 2014 Feb;17(2):269-79. doi: 10.1038/nn.3614. Epub 2013 Dec 15. PMID: 24336151; PMCID: PMC4004172.

Anti NeuN was validated for mouse by Ma KG, Hu HB, Zhou JS, Ji C, Yan QS, Peng SM, Ren LD, Yang BN, Xiao XL, Ma YB, Wu F, Si KW, Wu XL, Liu JX. Neuronal Glypican4 promotes mossy fiber sprouting through the mTOR pathway after pilocarpine-induced status epilepticus in mice. Exp Neurol. 2022 Jan;347:113918. doi: 10.1016/j.expneurol.2021.113918. Epub 2021 Nov 5. PMID: 34748756.

Additional validation information for all antibodies can also be found in the manufacturer's website.

Eukaryotic cell lines

Policy information about cell lines

Cell line source(s)	Both the HEK293T and BHK21 cell lines were purchased from ATCC (CRL-3216 and CCL-10 respectively)
Authentication	The cell lines used were not authenticated.
Mycoplasma contamination	The cell lines were periodically tested for mycoplasma, with no contamination detected.
Commonly misidentified lines (See ICLAC register)	No commonly misidentified cell lines were used in this study.

Animals and other organisms

Policy information about studies involving animals; ARRIVE guidelines recommended for reporting animal research

Laboratory animals	In addition to wildtype C57BL6-J mice, the following transgenic mouse lines were used:<br><br>Ai9 - B6.Cg-Gt(ROSA)26Sortm9(CAG-tdTomato)Hze/J Jackson labs (H. Zeng) 007909<br>GAD1-EGFP - Not deposited. K. Obata and Y.Yanagawa<br>RCE-FRT - Gt(ROSA)26Sortm1.2(CAG-EGFP)Fsh/Mmjax Jackson labs (G. Fishell) 32038<br>Prox1-cre - Tg(Prox1-cre)SJ32Gsat/Mmucd MMRRC (N. Heintz) 036644-UCD<br>KA1-cre - C57BL/6-Tg(Grik4-cre)G32-4Stl/J Jackson labs (S. Tonegawa) 006474<br>Dlx5/6-FlpE - Tg(mI56i-flpe)39Fsh/J Jackson labs (G. Fishell) 010815<br>Ctgf-2A-dgCre - B6.Cg-Ccn2tm1.1(folA/cre)Hze/J Jackson labs (H. Zeng) 028535<br>Ai32 - B6.Cg-Gt(ROSA)26Sortm32(CAG-COP4*H134R/EYFP)Hze/J Jackson labs (H. Zeng) 024109<br>Ai40 - B6.Cg-Gt(ROSA)26Sortm40.1(CAG-aop3/EGFP)Hze/J Jackson labs (H. Zeng) 021188<br>RosaDTA - B6.129P2-Gt(ROSA)26Sortm1(DTA)Lky/J Jackson labs (R. Locksley) 009669<br>In experiments, both male and female mice were used from all of these aforementioned lines, with ages ranging from 1-6 month old animals.
Wild animals	The study did not involve wild animals
Field-collected samples	The study did not involve samples collected in the field
Ethics oversight	The study was conducted under the following ethics approvals:<br>Bundesministerium für Wissenschaft, Forschung, und Wirtschaft of Austria (A. Haslinger, Vienna; BMWF-66.018/0010-WF/V/3b/2015; BMWFW-66.018/0008-WF/V/3b/2018).

Note that full information on the approval of the study protocol must also be provided in the manuscript.

Flow Cytometry

Plots

Confirm that:

☐ The axis labels state the marker and fluorochrome used (e.g. CD4-FITC).

☐ The axis scales are clearly visible. Include numbers along axes only for bottom left plot of group (a 'group' is an analysis of identical markers).

☐ All plots are contour plots with outliers or pseudocolor plots.

☐ A numerical value for number of cells or percentage (with statistics) is provided.

Methodology

Sample preparation	FACS analysis was used to determine viral titers of RVdG-CVS-N2c particles. For this purpose, HEK-TVA cells transduced at varying viral MOIs were resuspended 3 days post transduction using Trypsin C, fixed in PFA 4 % for 15 minutes, which was subsequently washed 2 times and replaced with PBS prior to analysis.
Instrument	BD Aria III sorter
Software	FACS Diva 6.1.3
Cell population abundance	The sample contained a pure population of HEK-TVA cells, with purity achieved by application of the strong antibiotic puromycin, against-which this cell line is immune.
Gating strategy	Preliminary gates were set at 50-150x10^3 and 100-250x10^3 for SSC and FSC respectively, encompassing the main population of cells. For quantification of the fluorescent moiety, the gate was set to exclude >99.9 % of cells of a non-transduced population.

☐ Tick this box to confirm that a figure exemplifying the gating strategy is provided in the Supplementary Information.
